# Progress, but at the Expense of Male Power? Institutional Resistance to Gender Equality in an Irish University

**DOI:** 10.3389/fsoc.2021.696446

**Published:** 2021-07-26

**Authors:** M. Hodgins, P. O’Connor

**Affiliations:** ^1^ Discipline of Health Promotion, National University of Ireland, Galway, Ireland; ^2^ Professor Emeritus Sociology and Social Policy, University of Limerick, and Visiting Professor, Geary Institute, University College Dublin, Dublin, Ireland

**Keywords:** institutional resistance, gender equality, university, Irish, associate professor, internal promotion, male power

## Abstract

Gender equality is a whole-organization endeavor. Building on Agócs (Journal of Business Ethics, 1997, 16 (9), 917–931) concept of institutionalized resistance this article undertakes a feminist critique of policy and practice around internal promotions to the equivalent of Associate Professor level in one Irish university (called the Case Study University). This university was selected because of its low proportion of women in senior academic positions. The methodology is a single case study design, employing documentary analysis, including secondary data. Since 2013 the proportion of women at Associate Professor in the Case Study University increased significantly (bringing them close to the national average): this being associated with increased transparency, with the cascade model in the background. However, men’s “chances” have varied little over time and at 1:4 are the highest in Irish universities. This article uses Agócs (Journal of Business Ethics, 1997, 16 (9), 917–931) stages of institutional resistance to show that while some changes have been made, ongoing institutionalized resistance is reflected in its failure to accept responsibility for change as reflected in its refusal to challenge the “core mission” and restricting the focus to “fixing the women”; and its failure to implement change by focusing on “busy-ness” which does not challenge power and colluding with foot-dragging and slippage in key areas. It is suggested that such institutional resistance reflects the enactment of hidden or stealth power. The article implicitly raises questions about the intractability and the covertness of men’s power and privilege and the conditions under which women’s “chances” are allowed to improve, thus providing insights into the extent and nature of institutional resistance.

## Introduction

Gender equality is a whole-organization endeavor reflecting the structure and culture of higher educational institutions (O’Connor, 2020a). The appointment in the past six months of women at Presidential/Rector level in four of the ten Irish public universities is historic, since no woman held that position in the previous 429 years. However, even after several national policy initiatives since 2014 (Athena SWAN^1^; Expert Group Review; Gender Equality Taskforce; Senior Academic Leadership Initiative; research funding agency initiatives and those around sexual harassment: O’Connor and Irvine, 2020) limited change of 1–2% per year has occurred in the proportion of women at full professorial level.

The aim of this article, which is focused at the organizational level, is to undertake a feminist critique of procedures and processes for internal promotion to the equivalent of Associate Professor level in one Irish university. This university was selected because its proportion of women in senior academic positions was particularly low (Higher Education Authority, 2017, 2016–2020). Hence the expectation was that it would provide insights into institutionalized resistance (Agócs, 1997). The university will be referred to as the Case Study University. The methodology is one of single case study design employing documentary and secondary data analysis. Specific documents are referred to by their type and date but not the name of the university (e.g. Commissioned Report^2^, 2016).

We outline the key concepts; methodology; main characteristics of the Case Study University; policy and procedures for internal promotion to Associate Professor; discuss stages of institutionalized resistance as reflected in a refusal to accept responsibility for dealing with change and the failure to implement change.

### Power and Institutional Resistance in Higher Educational Institutions

Power is required to meet organizational goals. In universities, with the impact of managerialism, there is an increasing centralization of power in the President/Rector and/or in the legitimacy of such centralization (Blackmore and Sachs, 2007; Deem et al., 2008; Lynch et al., 2012; O’Connor, 2014; O’Connor et al., 2019). In this context, many university structures (such as Executive Committee) become largely advisory, while others (such as Academic Council) are limited by lack of access to resources. Similarly, the power of positions such as Vice Presidents or Deans is reduced since these become fixed term Presidential appointments, where occupants serve at the pleasure of the President.

It is increasingly recognized that power in many organizations, including those in higher education, is gendered. Thus, a substantial body of research shows that women are under-represented in senior positions, with power being embodied at senior levels in men, enacted in male dominated structures, and characterized by a culture that facilitates masculinist priorities, lifestyles and relationships, with gender an organizing feature of these structures (Acker, 1990; Sinclair, 2005; Martin, 2020; O’Connor, 2020a).

Much of the early discourse on power explores overt, visible power. We are particularly interested in hidden power (Lukes, 1974; Gaventa, 1980) also referred to as stealth power; power that “operates covertly and panoptically” (Webb, 2008: 127; see also O’Connor et al., 2019). This enables us to transcend a post-heroic (Collinson, 2019) leader/follower paradigm. It reflects a recognition that those holding formal positions of power, can, through altering structures and shaping the organizational culture, exert hidden or stealth power by setting agendas, strike deals which limit others’ decision-making, prevent certain conflicts being vocalized, or define problems as individual rather than organizational.

Since universities are gendered, the exercise of this power is also gendered. It is enacted by formal male dominated masculinist structures which can predetermine regulations and practices regarding recruitment and promotion and therefore limit access to senior positions. This formal exercise of hidden or stealth power is supported by informal practices, which have been referred to as micropolitical practices (Morley, 2006; van den Brink et al., 2010; O’Connor, 2020b). These include “the strategies and tactics used by individuals and groups in an organization to further their interests” (van Den Brink et al., 2010: 25). They may be reflected in day-to-day interaction or in evaluation processes such as recruitment or promotion. They include gendered devaluation, stereotyping, procedural subversion, sponsorship, inbreeding etc. (Martin, 2003; O’Connor 2020b). Given the male dominated structure and culture in universities, even if these micropolitical practices are not overtly gendered, they are likely to favor men.

This gendered reality is obscured by legitimating discourses in universities globally. Such discourses “provide normative justifications for existing policies and practices through which they are seen as appropriate, reasonable, and fair and are consequently more readily accepted” (Tyler, 2005: 211). Legitimating discourses are social constructions which justify the status quo and reflect a construction of gender as a “primary way of signifying (and naturalizing) relations of power and hierarchy” (Mackay et al., 2010: 580). They include gender neutrality, excellence, choice, biological essentialism, and a depoliticized intersectional discourse (O’Connor and White, 2021). Such legitimating discourses typically either deny the existence of gender inequality or frame it as an individual “problem”. Even in so far as gender equality is recognized, strategies to deal with it frequently focus on individual women, with the implicit assumption that if women were more confident, better time managers, more political, made more appropriate life choices in effect if they were more like men-gender inequality in universities would not be a problem. Through the use of these legitimating discourses, powerful actors justify the underrepresentation of women in senior positions and other manifestations of gender inequality as “natural”, “inevitable”, women’s “choice” or inadequate “excellence.” These legitimating discourses can also be supported by micropolitical practices. Organizational factors are ignored, including evidence that the construct of excellence in higher education is frequently tautological, contested or reflects situationally specific masculinist criteria (Nielsen, 2016; van Den Brink and Benschop, 2012; O’Connor and Barnard, 2021). Thus the structures and culture of universities created by those in positions of power, through the use of hidden or stealth power, reflect institutionalized resistance to gender equality (Agócs, 1997).

The difficulty of creating gender equality in universities is well documented, (e.g. Burkinshaw and White, 2017; O’Connor, 2020a), with institutionalized resistance theorized as fundamental to understanding diluted, slow or no implementation of gender equality measures (Agócs, 1997; Benschop and Verloo, 2006; Lombardo and Mergaert, 2013; Powell et al.; Peterson et al., 2021; Strategaki, 2005; Smolovic Jones et al., 2020). Since gender equality challenges a powerful patriarchial order, such resistance is not unexpected. It takes many forms, overt and covert, actively or passively exercised (the latter through non-action and indifference). Gender equality can become difficult to challenge and can become more hidden and oblique, leading to a complex “dance” between overt and covert resistance (Smolovic Jones et al., 2020). However such distinctions may be less relevant in the noisy, contradictory world of organizations (Fleming and Spicer, 2008).

Here the focus is on institutionalized resistance, defined as “a pattern of organizational behavior that decision makers in organizations employ” (Agócs, 1997: 918). It can become “embedded in and expressed through organizational structures and processes of legitimation, decision making and resource allocation”. (Agócs, 1997: 919) claims that much organizational change literature is power-blind in that it fails to “address the ways in which gender and racial equality are built into the structures and cultures of organizations”. Institutional resistance to gender equality is often enacted covertly by those with power in the organization, who see gender equality as a challenge to their power and the privileged position they hold as a result of that power. Change driven solely by external factors such as national policy is likely to be resisted by them, if they see themselves as having something to lose from a change is the status quo. However, without such external pressure it is difficult for initiatives to get traction. Nowhere is this more evident than in the case of almost exclusively male senior management being required to share that power with women.

Agócs (1997): 48 typology of resistance identifies a sequence of four stages viz. denial of the need for change; refusal to accept responsibility for dealing with that change; refusal to implement change that has been agreed, and actions to dismantle change. Studies of resistance to transformative gender mainstreaming in public administration identify denial of the need for change and refusal to accept responsibility for change (Lombardo and Mergaert, 2013). Smolovic Jones et al. (2020) in a study of the British Labor party, identifies idealistic notions of absolute meritocracy combined with micropolitical practices involving localized decision-making, and the unreflexive enactment of long-established norms about suitable candidates for appointment as institutional resistance.

Within higher education, denial of the need for change as reflected in denial of the credibility of the message was evident in a case in 1989 (The Chilly Editorial Collective, 1995, as cited by Agócs, 1997) where a study by female faculty members involving discriminatory decision making around appointments and sexual harassment, was “publicly attacked by the University president, provost and several influential faculty members”. Two further internal reports which found high levels of discrimination and inequality, were neither responded to nor acknowledged by the university authorities (Agócs, 1997: 51). Peterson et al. (2021) also found evidence of institutional resistance to gender equality in Swedish and Portuguese higher education institutions. As in Van den Brink’s, (2015) study it was reflected in challenging the data and hence denial of the existence of gender inequality. Peterson et al. (2021) documented change agent’s perception of a refusal to take responsibility for change (including blaming the victims) combined with a strong commitment to what was seen as the preservation of a gender-neutral meritocracy. Powell et al. (2018) found that in their Swedish university study, the way gender equality was formulated reflected a discourse that women were the cause of the problem, reflecting a refusal to accept responsibility for change.

Peterson et al. (2021) also highlighted a refusal to allocate appropriate resources for gender equality as a reflection of institutional resistance. That was also noted in Temitope Igiebor’s (2021) work, which also highlighted policy silences, the absence of sanctions and the existence of gendered micropolitical practices inhibiting the implementation of gender equality policies in Nigerian higher educational institutions. Thus it is clear that similar patterns of institutional resistance to gender equality have been identified across a geographical spread of higher educational institutions, and that these broadly reflect Agócs (1997) types of institutional resistance.

We undertake a feminist critique of procedures and processes, looking at internal promotions to the equivalent of Associate Professor level in one Irish university. Building on Agócs typology we focus here on institutionalized resistance in the Case Study University, at the second and third stages, viz; the refusal to accept responsibility for dealing with change, and the refusal to implement change. We find evidence of a refusal to accept responsibility for change reflected in an unwillingness to effectively implement change in core institutional values and in the requirement for disadvantaged groups to change (“fix the women approach”: O’Connor, 2014; Burkinshaw and White, 2017). With regard to the refusal to implement change, we find evidence of displacement activity involving busy-ness around “soft actions” which leave power structures untouched, as well as foot dragging and slippage in a number of areas.

Theoretically then, the contribution of the article lies in enhanced understanding of institutionalized resistance to gender equality.

## Methods and Materials

The feminist critique of policy and procedures around internal promotion to Associate Professor or equivalent in one Irish university undertaken here is alert to the gendering of practices which may advantage men and disadvantage women, even if this is unintentional (Bensimon and Marshall, 2003: 338). The Case Study University was selected because the equation of power and position with masculinity was very deeply embedded there (Commissioned Report, 2016; Internal Report on Promotions, 2014: also Table 1).

**TABLE 1:** Percentage of female senior academics in Irish Universities 2013–19.

		Dec-13	Three year average 2013–15	Three year average 2016–18	Dec-19
ALL 7 Universities	Prof.	19%	19%	24%	26%
	Assc. Prof.	26%	27%	34%	37%
	SL	35%	35%	39%	39%
University A	Prof.	23%	23%	30%	29%
	Assc. Prof.	17%	17%	37%	34%
	SL	33%	33%	38%	36%
University B	Prof.	14%	16%	26%	31%
	Assc. Prof.	45%	43%	43%	48%
	SL	38%	34%	38%	36%
University C	Prof.	16%	18%	21%	24%
	Assc. Prof.	25%	29%	39%	39%
	SL	33%	29%	34%	37%
University D	Prof.	17%	19%	27%	31%
	Assc. Prof.	24%	29%	44%	51%
	SL	33%	35%	43%	42%
University E	Prof.	20%	20%	24%	24%
	Assc. Prof.	27%	29%	29%	33%
	SL	38%	38%	40%	40%
University F	Prof.	31%	31%	31%	28%
	Assc. Prof.	17%	17%	37%	41%
	SL	39%	39%	44%	39%
Case Study University	Prof.	14%	13%	13%	18%
	Assc. Prof.	13%	11%	18%	26%
	SL	30%	31%	40%	41%

Source: Higher education authority, 2016, 2017, 2108, 2019 2020; also internal report on promotions 2014.

The methodology is a single case study, in which the case is an institution, a methodology suitable for complex issues drawing on in-depth analysis of one case, while permitting transferability to other contexts (Simons, 2009). The methods involve documentary analysis (Bowen, 2009), including secondary data analysis (Follmer et al., 2012). The focus of that analysis is publicly available documents pertaining to gender equality in general in the Case Study University, with specific reference to internal promotion to the equivalent of Associate Professor. The analysis is thematic (Braun and Clarke, 2006), supplemented as appropriate by content analysis (Elo and Kyngas, 2008). In an attempt to ensure maximum rigor while maintaining anonymity, specific documents are referred to in this article by their type and date but not by the name of the university. These documents include:

1) Higher Education Authority institutional staff profiles by gender
2) Policy documents pertaining to promotional competitions between 2013 and 2020^3^
3) Athena SWAN institutional submissions
4) Institutional Gender Action Plan 2019 and review of its implementation
5) Reports commissioned by the university relating to gender equality such as Internal Report on Promotions (2014) and Commissioned Report (2016)
6) Case Study University web pages–2019–2021

Obviously, our approach raises issues related to access to data, bias etc. However, no research is bias-free, and awareness of potential sources of bias can enable critical reflection (Smith and Noble, 2014). Furthermore, an insider can provide insights unobtainable in any other way. Such work can be seen as a feminist activist reaction to the status quo: potentially “an act of transformational resistance” (Liu and Pechenkina, 2016: 191).

A good deal has been written about the role of feminist activists in initiating change within the masculinist male dominated contexts of academia (Bendl and Schmidt, 2012; Bendl et al., 2014; O’Connor, 2019). In such contexts, feminist agency may be reflected in a critique of policies and procedures through helping to “name, analyze, and think strategically about institutionalized resistance” (Agócs, 1997: 917). [Meyerson and Scully (1995: 586)] put forward the concept of “tempered radicals” individuals who identify with and are committed to their organizations, and are also committed to a cause, community or ideology that is fundamentally different from, and possibly at odds with, the dominant culture of their organization. They are inside/outsiders, people who are ambitious for their organization but also want it to change. Undertaking this work from inside an organization (the situation of one of the authors) poses both personal and intellectual challenges. In that context a second person can provide both support and perspective.

There is anecdotal evidence that, other than in a system where inbreeding dominates, (Cruz-Castro and Sanz-Menéndez, 2010; Montez-Lopez and O’Connor, 2019), external women are more likely to be appointed at senior level than internal ones. The appointment of three female Presidents (from Germany, United Kingdom and Finland) to Irish public universities in the past six months illustrates this phenomenon. It implicitly suggests that internal processes are problematic and prey to gendered micropolitical practices. Hence, this article, unlike much of the other work in the area, looks at internal promotional processes at Associate Professor or broadly equivalent level.

## Results

### Main Characteristics of the Case Study University

The Case Study University is a long established publicly funded University with just under 19,000 students and 2,000 staff; a suite of undergraduate and postgraduate programs, and several research institutes and centers. It was ranked in the top 260 of The World University rankings and in the top 1 per cent of universities worldwide on the Quacquarelli Symonds World University Rankings (Case Study University web 2021). There are 21 Schools in four Colleges, with disciplinary leaders in each school (a purely honorofic position). It has, like other Irish universities, experienced the shift to neoliberalism, with elements of collegiality (as reflected in election rather than appointment of line management) persisting up to recently. It commissioned a number of internal and external reports on gender equality from 2010 onwards (Internal Report on Distribution of Gender and Grade 2010; Internal Report on Academic Career Advancement 2011; Internal Report on Promotions 2014; Commissioned Report 2016), arguably reflecting Agócs (1997) tendency to try to deny the problem, including an external report (Commissioned Report, 2016) which firmly closed that option by noting that:

Many factors contribute to [Case Study University’s] poor performance on gender equality relative to the other universities in Ireland, which as a whole, perform relatively poorly on gender equality compared to many other European countries. The key factor is the culture within academia generally and in [Case Study University] in particular, which is based on gendered notions of what constitutes success and excellence. Changing this culture represents a major leadership challenge because it comprises an interlocking set of goals, roles, processes, values, communications practices, attitudes and assumptions (Commissioned Report, 2016: 25).

The Case Study University received an Athena Swan Institutional award (bronze) three years after its initial application. Three of its 21 Schools have secured a Bronze award (2017–2018) (Case Study University web, 2021).

### The Gender Profile of the Case Study University

The President and the Deputy President are both male, as all their predecessors have been. The gender profile of the official decision-making structures was poor in 2015, with women constituting 25% of Executive Management, 20% of Academic Council and 43% of Governing Authority (Higher Education Authority, 2019). This has improved, with women now constituting 44% of those on Executive Management, 48% of Academic Council and 43% of Governing Authority (Higher Education Authority, 2020). A low proportion of women are still in line management positions. There were no women Deans from 2016 to Jan 2020, with 14% being women (i.e. one person) between 2014 and 2016 (Athena SWAN application, 2017: 52). Women make up a larger proportion of Heads of School, although this proportion has also fallen slightly since 2014: from 44% in 2014 to 40% in 2021 (Internal Committee Audit Report, 2020). These posts were filled by election but since 2019 are overwhelmingly filled by internal appointment. It remains to be seen what impact this will have on their gender profile in the future.

The academic career structure in the Case Study University is broadly similar to other Irish universities with four main points on the academic scale: lecturer to (full) professor (Grade A: European Commission, 2019). Full professor is not a promotional grade, and is publicly advertised and filled through external competition and tied to formal managerial responsibilities. The Case Study University differs from other universities in that rather than having a specific Associate Professor grade, there is a post called a personal professor, paid without increments at roughly half way along the salary scale for the full professor post. This title in itself is unhelpful since it can be seen as eliciting a gendered stereotype and is a significant step up from the senior lecturer grade (Commissioned Report, 2016). Indeed, between 2009 and 2015, 80% of the completed applications for this position were from men (Commissioned Report, 2016). The Higher Education Authority^4^ assigns it to the Associate Professor category and this is the practice adopted here.

The Higher Educational Authority figures show that in December 2019, women made up 26% of those in Associate Professor positions in the Case Study University, increasing to 29% by December 2020 (Internal Progress Report, 2021), compared with an average of 37% across all then seven Irish universities. The increase occurred much later in the Case Study University (starting 2016–18) and from a lower base, bringing it closer to the national average (see Table 1). However, the extent of the change, and its limitations become obvious when we realize that in the Case Study University, women had a 1:42 “chance” of the broad equivalent of an Associate Professorship in 2013–15, improving significantly by 2019 to 1:13 (compared with 1:18 to 1:11 across all these universities in the same period: see Table 2).

**TABLE 2:** Women’s and men’s “chances” of securing a professorial position Irish Universities (Rounding to nearest whole number).

		Women’s “chances”			Men’s “chances”		
		Three year average 2013–15	Three year average 2016–18	Dec-19	Three year average 2013–15	Three year average 2016–18	Dec-19
ALL 7 Universities	Prof.	1:25	1:14	1:13	1:2	1:5	1:5
	Assc. Prof.	1:18	1:13	1:11	1:8	1:7	1:7
University A	Prof.	1:12	1:8	1:9	1:6	1:5	1:4
	Assc. Prof.	1:21	1:7	1:8	1:7	1:5	1:5
University B	Prof	1:21	1:14	1:11	1:5	1:6	1:6
	Assc. Prof.	1:8	1:8	1:6	1:8	1:8	1:7
University C	Prof	1:22	1:19	1:17	1:6	1:6	1:6
	Assc. Prof.	1:23	1:15	1:17	1:11	1:10	1:12
University D	Prof	1:18	1:19	1:16	1:5	1:6	1:7
	Assc. Prof.	1:27	1:25	1:24	1:11	1: 17	1:23
University E	Prof	1:12	1:10	1:11	1:4	1:4	1:4
	Assc. Prof.	1:14	1:15	1:13	1:6	1:7	1:7
University F	Prof	1:11	1:11	1:11	1:7	1:6	1:5
	Assc. Prof.	1:39	1:10	1:7	1:10	1:6	1:5
Case Study University	Prof.	1:28	1:29	1:24	1:5	1:5	1:5
	Assc. Prof.	1:42	1:21	1:13	1:5	1:5	1: 4

Source of data: Higher Education Authority, 2016, 2017, 2108, 2019 2020.

Men’s “chances” of an Associate Professorship across this same time period varied little over time and across universities (Table 2). Indeed, in four of the seven universities then in Ireland they increased between 2013 and 2019, with men in the Case Study University having the highest “chance”, at 1:4 in 2019. This implicitly raises questions about the intractability of men’s privileging and the conditions under which women’s “chances” improve.

There is a wider pattern of under-representation of women in senior positions in the Case Study University. It has consistently had the lowest proportion of women at full professorial level in any Irish university since 2013 (Table 1). This reluctance to appoint women to senior positions has also been evident in the administrative area where only 24% at those paid E106,000 p. a. were women as compared with 38% across all universities (Higher Education Authority, 2020).

Thus, although there are sectoral issues as regards the consistency of men’s privileging, there are particular questions as regards the Case Study University, where, despite improvement and effort, men’s “chances” remain three times higher than women’s “chances” at the equivalent of Associate Professor level.

### Policy and Procedures for Promotion at the Equivalent of Associate Professor

Personal professorial posts in the Case Study University (categorized as Associate Professor by the Higher Education Authority and hence here) are internal promotional posts secured on the basis of scholarly achievement and suitability. There is no limit to the number of appointments that the board can recommend, with applicants being ostensibly assessed against a normative standard rather than competing with each other (Internal Report on Promotions, 2014: 7). The procedures changed relatively little between 2009 and 2020 (The last call under the policy discussed here was February 2020).

To be eligible to apply for promotion to this position on the regular track, applicants must hold a senior lectureship and have reached the top of the salary scale (i.e. at least four years). The procedure includes three stages. Firstly, applications are assessed by the 14–16 person promotion board (14 up to 2016: Athena SWAN application, 2017) including the President and Deputy President as ex-officio members. (Seven or more members can in practice make that decision). Reflecting the continuing collegial ethos of the university, the other members are elected for five years. Up to January 2016, the gender profile of this internal board was 11 men and two women (Internal Report on Promotions, 2014: 9). Given the well-recognized importance of homosociability (Grummell et al., 2009), this raises legacy issues involving the extremely low “chances” of women being promoted from 2016 to 2018.

By 2018, the panel had been changed to include 50% of each gender, and the requirement for all members of the board to be at full professorship was dropped: with four of the eight men and three of the eight women being full professors. All board members have been required (since 2016) to undertake unconscious bias training (Case Study University web, 2021). However, the effectiveness of such training has been challenged internationally: “there is very little evidence that changes in implicit bias have anything to do with changes in a person’s behavior” (Oswald et al., 2015; Bartlett, 2017). Up to 2016 there was no scoring of candidates by the board; nor were there any strategies to eliminate conflicts of interests between the members and the applicants. It seems possible that applicants who had “paid forward” by doing favors for powerful members of the board were likely to be favored (O’Connor, 2020b).

Although officially both teaching and research were included as criteria, in practice promotion was primarily based on research output and income. More recently, c. 2018, a scoring system was introduced providing some transparency for individual candidates. However, it continues to favor research: of the 300 marks available, 200 are allocated to research and 50 each to teaching and learning and contribution to the university, school or community. A minimum score of 210 (70% overall) must be achieved to be recommended for promotion, and this must include a minimum score of 150 (75%) for research and scholarly standing, and a minimum of 20 (40%) in the two other areas. Given that women have 20–30% fewer publications (Rorstad and Aksnes, 2015; European Commission, 2019: 161), are less likely to be cited (Budrikis, 2020: 348), and to receive research grants (European Commission, 2019: 136), this scoring system advantages male candidates. Indeed 41% of female respondents in a survey of the scheme in the Case Study University felt they would never meet the level of achievement required, compared to 6% of the men (Internal Report on Promotions, 2014:13).

When a prima facie case for promotion is established, that board appoints the three members of the second Assessing Board, all external. There is no evidence of a requirement for this second board to be gender balanced. The assessors are asked for a written report on applicants’ research and scholarly standing informed by the 10 best papers submitted by the applicant. They are also asked whether the candidate would be suitable for promotion in the assessor’s university and to rank them in the top 5, 10 or 20% of applicants. It would be surprising if care was not taken by that board to select assessors who were likely to be supportive of those candidates favored by the board. There is no evidence that the board is aware that evaluations of male candidates tend to be higher than female candidates even in experimental situations (Moss-Racusin et al., 2012); or that letters of recommendation favor male candidates in terms of length, adjectives used and “doubt raisers” (Trix and Psenka 2003; Madera et al., 2009).

Competence is considered the most important criterion when evaluating candidates, however, while male applicants are assessed primarily on competence, female candidates are assessed on a wider range of criteria (Moscatelli et al., 2020) Thus, mothers are less likely to be evaluated positively than fathers (Gonzalez et al., 2019). In a potentially enlightened move, since 2015 applicants in the Case Study University were requested to document “leave taken”^5^ so as to allow the assessors to “adjust their expectations” providing “up to one year for each period of maternity leave” (Internal Promotional Documents 2019:17). This forces women to signal their parental status. It presupposes the existence of a supportive organizational culture. In its absence this information can evoke stereotypes and biases which disadvantage women.

There are additional potentially gendered requirements. Candidates are expected to make a clear and unequivocal case that they are currently performing at the higher level, and that they have the drive and capacity to continue performing at that level. Both may be problematic for female applicants, based on evidence that women who self-promote are more negatively evaluated than men who do so and are more vulnerable to a gender stereotyping backlash, since male stereotypes are more compatible with senior positions (Rudman and Glick, 2001:758). The tendency in women to play down achievements and for male and female assessors to view the achievements of men more favorably than those of women, raises further possibilities as regards gender bias.

The application process includes a commentary from the Head of School, who is expected to liaise with head of discipline, with oversight and sign-off by the Dean. This reflects a collegial ethos (Lynch et al., 2012), giving them (predominantly men), an opportunity to influence the process. Given informal practices such as sponsorship and homosociability (O’Connor, 2020b), this is likely to advantage men and disadvantage women.

The third stage involves the consideration of these reports by the promotion board and its recommendation to Governing Authority. It seems highly possible that “horse trading” (O’Connor and O’Hagan, 2016) occurs between the members of that board so that candidates favored by its most powerful members are recommended for promotion.

Additional routes were identified in 2013: namely “fast track” and leadership (Athena SWAN application, 2017: 43). The fast track category is an “exceptional” provision, which permits exemption from the top of the salary scale criterion, but requires external recognition through the award of an advanced higher degree (D. Litt, D.Sc., etc.) or “equivalent recognition”, as judged by the board (Internal Promotional Documents, 2019:1). Given men’s greater success at securing research grants (European Commission, 2019:115,116), the potential for gender bias in the judgment of “equivalent recognition”, and the lesser likelihood for women to be seen as exceptional (Van den Brink and Benschop, 2012), this route is unlikely to be helpful to women (men are more likely to attempt it: Commissioned Report, 2016).

The recommendation to increase the valuation of teaching (Athena SWAN application, 2017: 83), culminated in the leadership track. The grounds for selecting this track are “outstanding leadership” as evidenced by “projects, including strategic initiatives, which he/she has initiated and successfully implemented, the outcomes of those projects, and how those outcomes have impacted on the University’s performance” (Internal Promotional Documents, 2019). Given the under-representation of women in leadership positions in the Case Study University and the tendency for constructions of leadership to be gendered (Schein, 1998; Fitzgerald, 2018; Gandi and Sen, 2021), this also seems unlikely to be helpful to women (it is little used by either: Athena SWAN application, 2017: 44).

Unsuccessful applicants can appeal the process but only on procedural grounds, and only one appeal is permitted. The appeals committee comprises three people (men and women), as least two of whom will be professors or the equivalent of Associate Professor though women are under-represented in both positions (Internal Promotional Documents, 2019: 18).

A survey of senior lecturers in the Case Study University (with an overall response rate of 76%: including 82% of the women) identified a substantial lack of clarity, particularly among the women, about these criteria and procedures (Internal Report on Promotions, 2014). Thus, half of the women (and less than a quarter of the men) were not at all clear what was meant by demonstrating “a high and recognized international standard in scholarship and research”- a key issue since priority is given to research. Even higher proportions of the women (and a sizeable proportion of men) did not know how they could demonstrate a high level of achievement in teaching; while twice the proportion of women as men did not know what the board was looking for to “demonstrate a high and recognized international standard in academic leadership”. Even by 2018, almost one third of women felt they did not understand the promotion process and criteria (Internal Survey Report, 2019a).

Furthermore, although there was little difference in the qualifications, age, marital or family status of the men and women at senior lecturer level in the Case Study University in 2014, more men than women had been encouraged to apply for promotion (47% vs. 36%), mainly by Associate Professors (i.e. mainly men: Internal Report on Promotions, 2014: 9). This did not reflect their differential seeking of advice or length of time in the Case Study University (half of the women had worked there for at least 16 years compared with a third of the men: Internal Report on Promotions, 2014: 9). It may reflect homosocial micropolitical practices favoring men (Graves et al., 2019; O’Connor and Barnard, 2021). Overall although there have been some changes, an unambiguous focus on reducing gender inequality has been missing.

In February 2015, a Gender Equality Task Force (almost half of whom were external to the Case Study University) was established with a remit: to “advise the University on what measures it should take to develop effective gender equality” (Commissioned Report, 2016:9). It challenged the myths underpinning institutionalized resistance (Agócs, 1997): for example, the idea that the construct of excellence is gender neutral (Commissioned Report, 2016: 18) and that women are “the problem”.

It made 24 recommendations including the appointment of an adequately resourced Vice President of Equality, the first such appointment in an Irish university; the introduction of “mandatory gender quotas for all academic promotions and competitions” based on the flexible cascade model (i.e. a soft quota) with the number promoted being based on the number of eligible women at the grade below (Commissioned Report, 2016: 40), if necessary phased in over a maximum of two rounds of these competitions. Both of these recommendations were re-iterated at national level by the Expert Group (Higher Education Authority, 2016a) which recommended linking state funding to the gender profile of senior positions and a gender quota of 40 per cent women at full professorial level. The report on the Case Study University also recommended a review of its academic grading structure, including the Associate Professorship; the development of principles to underpin workload models (since women were more likely to be allocated administrative responsibilities: Misra et al., 2011); and “Detailed, specific exemplars of what constitutes excellence for the various areas of academic activity” (Commissioned Report, 2016: 41). There were other “softer” recommendations. Nevertheless, it was an attempt at structural and cultural transformation.

### Implementation of Policies: Transparency and the Cascade Model

From 2009 to 2015 there was little information and even less transparency about the Associate Professor process in the Case Study University (Internal Report on Promotions, 2014). Eight rounds of promotions took place involving 69 applications, of which only 14 (20%) were from women (Commissioned Report, 2016: 24). This could be seen as indicating a lack of confidence in the procedures and/or a low proportion of eligible women. The proportion of women in these positions increased from 10% in 2014 to 16% in 2016 (reflecting increases in STEMM: no data available on non-STEMM: Athena SWAN application, 2017: 24).

Following the 2016 Commissioned report, a Vice President was appointed and an office staffed (two persons). This office has since initiated three institutional committees with an equality focus, appointed a Vice Dean for Equality Diversity and Inclusion in each of the four Colleges, developed a Gender Equality Action Plan in 2019 and reviewed it (Internal Progress Report, 2021) and put in place an external advisory group. Gender balance on committees has also been monitored and reported.

From 2017 to 2020 inclusive, 43% of those who applied for an Associate Professorship were promoted (29/68), with roughly a fifth of the men and women who applied being successful (13/68 women V 15/68 men: Table 3). The proportion of women promoted to Associate Professor, with the exception of 2017, exceeded that in the senior lecturer pool (Table 1). The gains for woman were more marked and consistent after 2018, and placed the Case Study University close to the national average (Table 1). This change coincided with improved transparency, including a scoring system and making public the composition of the promotions panel. The number, gender and discipline of applicants at the various stages and ultimately of those promoted was not provided; nor were the applications of successful candidates made available; nor data on appeals.

**TABLE 3:** Promotions to equivalent of Associate. Prof. in the Case Study University: 2017–2020.

Year	2017	2018	2019	2020
Total applicants	15	29	13	11
Applicants who got through first stage	7 male, 3 female	16 male, 11 female	4 male, 4 female	5 male, 4 female
Number promoted	2 male, 0 female	8 male, 7 female	3 male, 3 female	2 male, 3 female

Source: The Case Study University Equality Office.

Gender quotas based on a flexible cascade model are in the Gender Action Plan (2019), but are not explicitly referred to in the promotional documentation. This could be to avoid criticism (Agócs, 1997: 55). However, an explicit, transparent cascade policy could encourage female applicants and prevent slippage (although it may have the opposite effect in highly feminized professions).

Legacy issues such as the requirement to have reached the top of the senior lecturer (SL) salary scale before applying for an Associate Professorship on the regular track was not removed by the end of the scheme (February 2020) although this was recognized as an issue in 2014; listed for action in 2017 (Athena SWAN application, 2017: 44) and reappeared in the 2019 Gender Equality Action Plan.

The ongoing gap in men and women’s “chances” suggests that transparency alone (even with the background existence of the cascade model) is not sufficient (van den Brink et al., 2010).

## Discussion of Stages of Resistance

The aim of this article is to undertake a feminist critique of procedures and processes for internal promotion to the equivalent of Associate Professor level in one Irish university in order to provide insights into institutionalized resistance (Agócs, 1997). As stated previously, Agócs (1997: 48) identifies a sequence of four stages of resistance: denial of the need for change; refusal to accept responsibility for dealing with that change; refusal to implement change, and actions to dismantle change.

Although it is not the focus here, it is suggested that the first stage of institutional resistance was reflected in the historically extreme position of the Case Study University and its stubborn denial of the existence of gender inequality up to 2016. There have been some improvements since then, most notably the increase in women’s “chances” of promotion to Associate Professor from 1:42 in the 2013–15 period to 1: 13 in 2019. Yet, this current level is more than three times worse than men’s “chances”. We suggest that this reflects the second stage of Agócs typology of institutional resistance viz. an institutional failure to accept responsibility for dealing with change and (to some extent) the third stage viz. refusal to implement change.

We identify two sub-categories of the second stage viz. refusal to challenge what is seen as “core mission” of the university and “fixing the women”. These have been identified in other studies (O’Connor, 2014; Burkinshaw and White, 2017; O’Connor, 2020b; Peterson et al., 2021; Temitope Igiebor, 2021). We also identify two sub-categories of the third stage viz. a refusal to implement change as reflected in the displacement of energy away from tackling power inequalities into “busyness” and foot dragging and slippage in a number of key areas. These are particularly important in the Case Study University where there appears to be a willingness by those in formal positions of power to use stealth power to collude with such tactics and so avoid the need for fundamental structural and cultural change.

### Stage of Institutional Resistance: Failure to Accept Responsibility for Change

Based on Agócs (1997) schema, the second stage of institutional resistance is seen as including a refusal to challenge the “core mission” of the university and a focus not on fixing the university but on “fixing the women”.

#### Refusal to Challenge the Core Mission

In the Case Study University there is an uncritical assumption that a meritocratic approach involving excellence is completely free of gender bias, despite caveats expressed in the Commissioned Report (2016) based on Castilla and Benards (2010: 543) evidence that “when an organizational culture promotes meritocracy … managers in that organization may ironically show greater bias in favor of men over equally performing women”. The internal Associate Professor scheme in the Case Study University has continued to list criteria (albeit with some adjustment in their number or the weighting of one set against the other) based on the assumption that to change this would be to challenge the core mission of the institution (Agócs, 1997: 55). This can be seen as reflecting the exercise of hidden or stealth power by the power holders: the challenging of this being literally unthinkable.

There is now considerable evidence that the definition and operationalization of the construct of excellence is frequently gendered and tautological (Van den Brink and Benschop, 2012; Nielsen, 2016; Campbell, 2018; Ferretti et al., 2018; O’Connor and Barnard, 2021). Excellence has been seen as a rationalizing myth in academia used to legitimate evaluative decisions and to obscure gendered processes (O’Connor and Barnard, 2021). There is no recognition of this in the Case Study University. The criteria outlined in the promotional documentation since 2018 relate to “Research and Scholarly Standing”, “Teaching and Learning”, and “Contribution to School, College, University and Community. Most appear, at face value, to be appropriate to promotion (student feedback, teaching approach, research funding, scholarly standing, research leadership etc.) but the standards to be attained, and hence the scores, involve judgments that are highly subjective and therefore open to bias. Even for criteria where numerical benchmarks could be applied, subjective terms are employed (“evidence of consistent and continuing high-quality output of research publication in peer-reviewed journals, scholarly works”: Internal Promotional Documents, 2019: 3). Phrases such as such as “high level of achievement”, “maintaining theoretical currency” “evidence of scholarly contribution”, “recognition by peers” (Internal Promotional Documents, 2019; 2–4), appear with no benchmark or objective indictors despite the recommendation to specify the standard expected (Commissioned Report, 2016:41). These are all presented and applied as gender-neutral. The CVs of successful candidates, which could facilitate a gendered analysis, have also not been made available.

More fundamentally, the bias in favor of research ensures that men’s privilege is maintained. The evidence that men outperform women in this area is well established, yet this bias is embedded into the promotion scheme (European Commission, 2003). Women are also likely to carry heavier teaching and administrative loads (not least because the male dominated hierarchies endorse stereotypical views about women and devalue their skills and attributes), and this is seen as “natural” and inevitable.

Many of these processes also occur in other universities: and are arguably not unrelated to the low levels of variation in men’s “chances” in Irish universities (see Table 2). However, the targets set in the Case Study University reveal the desire to maintain male privilege. Thus, whereas nationally, there is a quota of 40% of the full professoriate to be women by 2024 (Higher Education Authority, 2016b) the Case Study University has considerably less ambitious targets: 28% by 2024–including both full professors and those in the broadly equivalent Associate Professor category (Case Study University web, 2019).

#### Restricting the Focus to “Fixing the Women”

In the case study’s Gender Equality Action Plan (Internal Progress Report, 2021) there is a good deal of reliance on an uncritical “fix the women” approach (O’Connor, 2014; Burkinshaw and White, 2017). This is seen by Agócs (1997) as indicating a failure to take responsibility for change.

There are 20 references to leadership in that Gender Equality Action Plan, all reflecting a deficit model (Burkinshaw and White, 2017), with women’s lack of skills being used as a legitimating discourse. Thus, there is a reference to making women serious candidates for promotion (Van den Brink and Benschop, 2012: 81) by coaching, mentoring and training them. There is also a reference to “a strong pipeline” at Associate Professor level (see Table 1) reflected in “the pool of women eligible for promotion to Professor in the coming years” (Internal Progress Report, 2021: 16). This implicitly rejects the idea that the low proportion of women reflects organizational factors and suggests that “the problem is women”, with the cause of women’s under-representation being framed in terms of individual’s deficits.

The Gender Equality Action Plan (Internal Progress Report, 2021) also refers to three separate leadership initiatives i.e. Academic Career Development Workshops, Aurora Leadership training, and Athena SWAN Leadership seminar series. “Successful” women leaders are presented as role models with structural difficulties obscured. There is an uncritical view of the impact of such programs. This is in contrast to [Manfredi et al. (2014): 54] which found that female alumni of its top management program who applied for senior management roles were more than twice as likely as their male counterparts to have been unsuccessful (22% vs. 8.5%).

Maternity is still seen as an aberration. Thus, “ramp-up post-maternity workshops” are provided for women returning from protected leave including maternity leave (Internal Progress Report, 2021: 20) with small grants available since 2016. These are helpful but do not deal with the underlying organizational cultural and structural problems, and can be seen as reflecting institutional resistance to systemic change.

### Institutional Resistance: Failure to Implement Change

Agócs (1997: 56) describes this third stage of resistance as involving overt claims of responding to a change message, but with no real change occurring. It includes failure to implement or enforce policy, failure to ensure accountability or delegation of responsibility for implementing change or failure to allocate the necessary resources for such work. While it would be untrue to say that no real change has occurred in the Case Study University, the slow rate of change compared to other Irish Universities (see Table 1) suggests that some elements of this stage of resistance are present.

Here it is seen as reflected in busy-ness which does not challenge power and in foot-dragging and slippage in a number of areas.

#### Busy-Ness Which does not Challenge Power

Here we focus on busy-ness involving de-politicized actions as a stage of institutional resistance. The Gender Equality Action Plan contains 89 actions. Neither power nor inequality is mentioned, “discrimination” only once, while “support” appears 34 times; “committee” 20 times. The references to gender are couched in sanitized terms such as “inclusion” and welcoming “diversity”. There is a strong impression that there is a preference for “safe” actions that do not challenge the power structures in the Case Study University. Implicit in this is a kind of de-politicized intersectional approach one that fails to recognize power inequalities and their implications (Indeed, in one College the Vice-Dean is of Equality, Diversity and Wellness, aligning equality with lifestyle).

The Internal Progress Report (2021:1) on the implementation of the Gender Equality Action Plan finds 41 actions completed, a third of which involve the setting up of committees, return of data to the Higher Education Authority, the creation of Athena SWAN structures and the Gender Equality Action Plan itself and reporting on its implementation. Many of the completed actions do not challenge power for example a video showcasing senior women in leadership roles, celebrating diversity events, roadshows for family leave entitlements, and the creation of parental support networks.

Although the proportion of women at Associate Professor has increased, reflected in the improvement of women’s “chances” (which at 1:13 are close to the national average of 1:11) the persistently high- and indeed increase in men’s “chances” in 2019 (at 1:4, they are now the highest, compared to a 1:7 average across all universities: See Table 2) point to institutional resistance.

The need to address gender equality in Case Study University as a deep gendered structural and cultural problem was first recognized in 2016 (Commissioned Report, 2016). Few of the actions to date have addressed this, with the possible exception of the creation of an office for equality and increased transparency (against the backdrop of the cascade model). However, this is concealed by the “busyness” of the actions in Gender Equality Action Plan.

#### Foot-Dragging and Slippage in a Number of Areas

Here we focus on foot-dragging involving the implementation of recommendations made over a number of years, including the introduction of a new typical Associate Professor grade***,*** the persistence of gendered requirements for promotion to the current equivalent of Associate Professor, legacy issues and the lack of progress on gender balance on committees and line management.

The introduction of a grade of Associate Professor similar to that in other universities was recommended in a report in 2016 (Commissioned Report, 2016: 14); it re-appears as a recommended action in the institutional application for an Athena SWAN award in 2017 (Internal Application Document 2017: 25) but is recorded as “delayed” in the progress report (Internal Progress Report, 2021: 17). This contrasts with the moving ahead of plans to consolidate the superior status and salary of full professor: a position where women are still very under-represented.

Foot dragging is reflected in the persistence of gendered requirements for promotion to the broad equivalent of Associate Professor, such as the requirement for applicants to make a clear and unequivocal case that they are performing at the higher level; and the maintenance of fast track and leadership additional routes, despite their unhelpfulness as regards gender equality. These requirements, and the failure to acknowledge or defend against micropolitical practices such as sponsorship and homosociability, all point to difficulties in implementing change in the gender profile of senior positions.

Failure to deal with legacy issues is also evident. Women have been under-represented at senior lecturer level in the Case Study University for many years (see Table 1) a situation which only started to improve in 2017 (Higher Education Authority, 2018). Retaining the requirement to wait four years until these women reach the top of the salary scale to apply for promotion to Associate Professorship is evidence of foot-dragging.

Further evidence of failure to implement change can be found in slippage regarding gender balance on committees, and in the ongoing low proportion of women still in line management positions. At the Case Study University there has only been one female Dean between 2014 and 2016 and none since then (Athena SWAN application, 2017: 52). The proportion of female Heads of School, while considerably higher at 44% in 2014 actually slipped to 40% in 2020 (Internal committee Audit Report, 2021). Similarly, whereas 66% of all committees were compliant with 40% gender balance in 2018, this dropped to 55% in 2019 and to 46% in 2020 (Case Study University web, 2021).

Slippage is also evident in the description of the very modest targets identified at professorial level as delayed (Internal Progress Report, 2021). Other interventions that might challenge men’s advantage are also identified there as “at risk”: including gender disaggregation of all personnel data; demonstrable experience of leadership in advancing gender equality for appointment to senior leadership roles; the development of a competency framework and a promotional scheme for professional and administrative staff (Internal Progress Report, 2021:10). It is suggested that these also reflect foot dragging and slippage and ultimately a failure to implement change and a reflection of the third stage of institutionalized resistance (Agócs, 1997). They can also be seen as a manifestation of the enactment of hidden or stealth power.

## Summary and Conclusion

The challenges in promoting gender equality in higher educational institutions have been identified in many studies (e.g. Benschop and Verloo, 2006; Lombardo and Mergaert, 2013; Burkinshaw and White, 2017; O’Connor, 2020a; Smolovic Jones et al., 2020; Powell et al.; Peterson et al., 2021). Building on Agócs (1997) conceptualization, the contribution of this article lies in an enhanced understanding of institutionalized resistance to gender equality as a manifestation of stealth power, reflected in a refusal to accept responsibility for dealing with change and the failure to implement aspects of a change agenda, both indicating an unwillingness to recognize that institutional transformation is required. We demonstrate how the analysis of documentation facilitates the identification of these sub-types of institutional resistance, as does the calculation of men’s chances in comparison to women’s chances, methods which could be applied in other organizations to reveal the extent and nature of institutional resistance to gender equality.

Gender inequality was identified in 2015 as a particular problem in the Case Study University (Commissioned Report, 2016), and this was the reason for its selection. In this article we have been particularly concerned with the procedures involved in internal promotion to the equivalent of Associate Professor and the related Gender Equality Action Plan (2019) and evaluation of its implementation (2021). It has been shown that women’s “chances” have improved dramatically: this coinciding with increased transparency in the process, and the flexible cascade model (i.e. soft quota).

However, in the Case Study University, the “normal” procedures and criteria are designed by men for men. This helps explain the low level of variation in men’s “chances”. The particularly masculinist culture in that university was reflected in women’s 1:42 “chance” of promotion to Associate Professor in 2013–2015; in the total lack of transparency in the procedures and in the very designation of the Associate Professor position as personal professor which served to create further difficulties for women and to embed male privilege. Residues of these remain: the net effect being that although women’s “chances” in the Case Study University have improved (and at 1:13 are close to the national average of 1:11) men’s have also increased and at 1:4, are now the highest in the country (compared to a 1:7 average across all Irish universities).

Despite an overt concern with the under-representation of women in senior positions, the masculinization of line management positions has continued and is being effectively ignored. There has been no attempt to see the problem as an organizational one and to challenge the “core mission” of the university but instead the focus is on “fixing the women”, reflecting Agócs (1997) second form of institutional resistance. There has been slippage in women’s representation on committees, in line management positions and in the implementation of policies in a number of areas, as well as foot dragging on a number of fronts, despite a large amount of “soft” activity (these being seen as reflecting Agócs, 1997 third form of institutional resistance). Thus, it is almost as if, losing ground on some fronts, institutionalized resistance ensured that it was gained on other fronts, through the exercise of hidden or stealth power.

It seems possible to conclude that in Case Study University (as indeed in all Irish universities) improvements in women’s position will only be accepted if men remain ahead of them. However, this pattern is heightened in the Case Study University, most recently as reflected in an attempt to consolidate the superior status and salary of the full professor; in the slow increases in the proportion of women at this level-and the effective acceptance of this slow pace as reflected in the sleight-of-hand foot dragging around the national quota of 40% of women at full professorial level by 2024. Thus, in the Case Study University although there has been some progress for women, it has not been at the expense of male power and privilege.

## Recommendations to Circumvent Institutionalized Resistance

Institutionalized resistance needs to be addressed at the institutional level. Exposing the way in which power operates in organizations, in particular how those who hold power can use it to frame interpreptations of the “problem” of gender inequality and circumscribe solutions to those problems by the exercise of hidden or stealth power through structures, legitimating discourses, processes and micropolitical behaviors is crucial. At the very least, the inevitability of institutional resistance needs to be surfaced and recognized. Only then can change agents start to dismantle it.

In particular the uncritical assumption that a meritocratic approach is gender neutral needs to be challenged. This has been identified in a number of studies. Since academic institutions can be relied on to robustly defend their activities as meritocratic, this will require innovative and creative approaches, beyond traditional information sessions. Drawing on dedicated resources such as the FESTA handbook on resistance to gender equality in academia (Saglamer et al., 2016) may be a useful entry point since it aims to facilitate a deeper understanding of institutional resistance, with practice-based, exemplary vignettes, presenting its causes, indicators and ways of dealing with it.

The significance of data was clearly acknowledged by the national Expert Group in their recommendations for year-on-year statistics on the percentages of women in senior positions across Irish Higher Educational Institutions (Higher Education Authority, 2016a). Although a limited approach, the availability of data can make a problem and the solution visible. The annual publication of gender profiles since 2016 has made resistance through denial of gender equality less viable. Collecting and publishing national data at a more granular level on individual institutions, including the gender breakdown of all stages of the applications, including withdrawals/resignations and retention, length of time in each position and the gender profile of principal investigators/Directors of Research Centers can help reveal fault lines and benchmark successes. The public availability of the CVs of successful candidates would be very helpful in such benchmarking. Monitoring women’s and men’s “chances” of securing senior posts, as we have done here, would a useful addition to institutional profiles. The role of the Higher Educational Authority is important in ensuring that the targets identified are compatible with national policy and that resources to achieve them are identified internally, with their achievement related to state funding.

The findings here are consistent with many other studies that reveal a reliance on “adding women to things” (e.g. Committees, interview boards) or “adding things to women” (e.g. CV writing or leadership skills). We strongly recommend moving the focus from women to institutional structures and processes that privilege men. Privilege and the mechanisms that maintain it must be exposed, if institutional resistance is to be successfully dismantled. Higher Education Institutions might benefit from training materials developed to surface white privilege (see for example McIntosh, 2010). Finally, case studies involving the structures, processes, procedures and leadership in those higher education institutions that exemplify best practice in this area would also be important in moving the issue forward.

## Data Availability

Publicly available datasets were analyzed in this study. While the data used in this study (documents) is publicly available, we are bound to protect the identify of the organization.

## Footnotes

1 Athena SWAN (AS) is a gender equality charter, which originated in the United Kingdom in 2005 and has been subsequently adopted/piloted in Australia, Canada, United States as well as in Ireland. Initially focusing on gender equality in the career-progression of women in science, technology, engineering, mathematics and medicine, it has been subsequently extended to include all disciplines as well as professional, technical and support staff. Awards are given at institutional and departmental level and in bronze, silver and gold. For a more detailed description, see O’Connor and Irvine (2020).

2 To protect the identify of the University, we have anonymized certain references, using generic descriptive titles rather than institutional names or authors. Full citations of all anonymized documents has been supplied in confidence to the Journal Editor.

3 A revised promotions process commenced in January 2020 (slightly overlapping with last call under older process) and is not included in this analysis

4 Higher Education Authority uses the term “Associate Professor” (Associate Professor) for comparability across institutions, although titles, terms and conditions vary somewhat between Universities. This data excludes the three new Universities created in 2020.

5 Time out for documented leave including: maternity; paternity; parental; sick/disability; and carer’s leave is discounted in the assessment of applications (Case Study University web, PDs, 2019)

## References

[B77] Acker J. (1990). Hierarchies, Jobs, Bodies: A theory of Gendered Organizations. Gend. Soc. 4 (1), 139–158. 10.1177/089124390004002002

[B1] Agócs C. (1997). Institutionalized Resistance to Organizational Change: Denial, Inaction and Repression. J. Business Ethics. 16 (9), 917–931. 10.1023/a:1017939404578

[B2] Bacharach S. B. Baratz M. S. (1962). The Two Faces of Power. Am. Polit. Sci. Rev. 56, 947–952. 10.2307/1952796

[B3] Bartlett T. (2017). Can We Really Measure Implicit Bias? Maybe Not, *the Chronicle Of Higher Education* . Available at: https://www.chronicle.com/article/Can-We-Really-Measure-Implicit/238807.

[B4] Bendl R. Danowitz M. A. Schmidt A. (2014). Recalibrating Management: Feminist Activism to Achieve Equality in an Evolving university. Br. J Manage. 25 (2), 320–334. 10.1111/1467-8551.12008

[B5] Bendl R. Schmidt A. (2012). Revisiting Feminist Activism at Managerial Universities. Equal Div Incl: Int. J. 31 (5/6), 484–505. 10.1108/02610151211235488

[B6] Benschop Y. Verloo M. (2006). Sisyphus' Sisters: Can Gender Mainstreaming Escape the Genderedness of Organizations? J. Gend. Stud. 15 (1), 19–33. 10.1080/09589230500486884

[B7] Bensimon E. M. Marshall C. (2003). Like it or Not. J. Higher Education. 74 (3), 337–349. 10.1080/00221546.2003.11780850

[B8] Blackmore J. Sachs J. (2007). Performing and Reforming Leaders: Gender, Educational Restructuring and Organisational Change. Albany, NY State: University of New York.

[B9] Bowen G. A. (2009). Document Analysis as a Qualitative Research Method. Qual. Res. J. 9 (2), 27–40. 10.3316/qrj0902027

[B10] Braun V. Clarke V. (2006). Using Thematic Analysis in Psychology. Qual. Res. Psychol. 3 (2), 77–101. 10.1191/1478088706qp063oa

[B11] Budrikis Z. (2020). Growing Citation Gender gap. Nat. Rev. Phys. 2, 346. 10.1038/s42254-020-0207-3

[B12] Burkinshaw P. White K. (2017). Fixing the Women or Fixing Universities: Women in HE Leadership. Administrative Sci. 7, 30. 10.3390/admsci7030030

[B13] Campbell L. D. Astrin J. J. DeSouza Y. Giri J. Patel A. A. Rawley-Payne M. (2018). The 2018 Revision of the ISBER Best Practices: Summary of Changes and the Editorial Team's Development Process. Biopreserv Biobank. 16, 3–6. 10.1089/bio.2018.0001 29393664 PMC5846567

[B14] Castilla E. J. Benard S. (2010). The Paradox of Meritocracy in Organizations. Administrative Sci. Q. 55 (4), 543–676. 10.2189/asqu.2010.55.4.543

[B15] Collinson D. L. (2019). “Critical Leadership Studies: Exploring the Dialectics of Leadership,” in What’s Wrong with Leadership? Improving Research and Practice. Editor Riggio R.E. (Abingdon: Routledge), 260–278.

[B16] Cruz-Castro L. Sanz-Menéndez L. (2010). Mobility versus Job Stability: Assessing Tenure and Productivity Outcomes. Res. Pol. 39 (1), 27–38. 10.1016/j.respol.2009.11.008

[B17] Deem R. Hilliard S. Reed M. (2008). Knowledge, Higher Education and the New Managerialism. Oxford: Oxford University Press.

[B18] Elo S. Kyngäs H. (2008). The Qualitative Content Analysis Process. J. Adv. Nurs. 62 (1), 107–115. 10.1111/j.1365-2648.2007.04569.x 18352969

[B19] European Commission (2003). *SHe Figures 2003*. Women and Science Statistics and Indicators. Brussels: European Commission Available at: https://op.europa.eu/en/publication-detail/-/publication/31442d26-88c7-42db-a985-b8d843517089/language-en.

[B20] European Commission (2019). SHE Figures 2018. Brussels: European Commission Available at: https://ec.europa.eu/info/publications/she-figures-2018_en.

[B21] Ferretti F. Pereira Â. G. Vértesy D. Hardeman S. (2018). Research Excellence Indicators: Time to Reimagine the 'making of'? Sci. Public Pol. 45 (5), 731–741. 10.1093/scipol/scy007

[B22] Fitzgerald T. (2018). Looking Good and Being Good: Women Leaders in Higher Education in Australian Universities. Education Sci. 8 (2), 54. 10.3390/educsci8020054

[B23] Fleming P. Spicer A. (2008). Beyond Power and Resistance. Management Commun. Q. 21 (3), 301–309. 10.1177/0893318907309928

[B24] Follmer A. Greenhoot A. Dowsett C. J. (2012). Secondary Data Analysis: An Important Tool for Addressing Developmental Questions. J. Cogn. Development. 13 (1), 2–18. 10.1080/15248372.2012.646613

[B25] Gandhi M. Sen K. (2021). Missing Women in Indian university Leadership: Barriers and Facilitators. Educ. Management Adm. Leadersh. 49 (2), 52–369. 10.1177/1741143219896048

[B26] Gaventa J. (1980). Power and Powerlessness. Quiescence and Rebellion in an Appalachian Valley. Chicago: University of Illinois Press.

[B27] Gender Equality Taskforce (2018). Gender Action Plan 2018-2020. Dublin: Higher Education Authority.

[B28] Graves A. Rowell R. Hunsicker E. (2019). An Impact Evaluation of the Athena Swan Charter. Ortus, Loughborough University Available at: https://www.ecu.ac.uk/wp-content/uploads/2019/08/Athena-SWAN-Impact-Evaluation-2019.docx.

[B29] Grummell B. Devine D. Lynch K. (2009). Appointing Senior Managers in Education: Homosociability, Local Logics and Authenticity in the Selection Process. Educ. Management Adm. Leadersh. 37 (3), 329–349. 10.1177/1741143209102783

[B81] González M.J. Cortina C. Rodríguez J. (2019). The Role of Gender Stereotypes in Hiring: A Field Experiment. Eur. Sociol. Rev. 35 (2), 187–204. 10.1093/esr/jcy055

[B30] Higher Education Authority (2016a). National Review of Gender Inequality in Irish Higher Education Institutions. Dublin: Higher Education Authority Available at: https://hea.ie/assets/uploads/2017/06/HEA-National-Review-of-Gender-Equality-in-Irish-Higher-Education-Institutions.pdf.

[B31] Higher Education Authority (2016b). Higher Education Institutional Staff Profiles By Gender Dublin: Higher Education Authority Available at: https://hea.ie/assets/uploads/2017/06/Higher-Education-Institutional-Staff-Profiles-by-Gender.pdf.

[B32] Higher Education Authority (2017). Higher Education Institutional Staff Profiles by Gender. Dublin: Higher Education Authority Available at: https://hea.ie/assets/uploads/2017/07/HEA-Institutional-Staff-Profiles-Gender-July-2017-003.pdf.

[B33] Higher Education Authority (2018). Higher Education Institutional Staff Profiles by Gender. Dublin: Higher Education Authority Available at: https://hea.ie/assets/uploads/2018/01/Higher-Education-Institutional-Staff-Profiles-by-Gender-2018.pdf.

[B34] Higher Education Authority (2019). Higher Education Institutional Staff Profiles by Gender. Dublin: Higher Education Authority Available at: https://hea.ie/assets/uploads/2020/01/Higher-Education-Institutional-Staff-Profiles-by-Gender-2019.pdf.

[B35] Higher Education Authority (2020). Higher Education Institutional Staff Profiles by Gender. Dublin: Higher Education Authority Available at: https://hea.ie/assets/uploads/2019/07/Higher-Education-Institutional-Staff-Profiles-by-Gender-2020.pdf.

[B36] Liu H. Pechenkina E. (2016). Staying Quiet or Rocking the Boat? an Autoethnography of Organisational Visual white Supremacy. Equality, Divers. Inclusion: Int. J. 35 (3), 186–204. 10.1108/edi-08-2015-0067

[B37] Lombardo E. Mergaert L. (2013). Gender Mainstreaming and Resistance to Gender Training: A Framework for Studying Implementation. Nordic J. Feminist Gend. Res. 21, 4296–4311. 10.1080/08038740.2013.851115

[B38] Lukes S. (1974). Power: A Radical View. Basingstoke: Palgrave Macmillan. 10.1007/978-1-349-02248-9

[B39] Lynch K. Grummell B. Devine D. (2012). New Managerialism in Education: Commercialisation, Carelessness and Gender. Basingstoke: Palgrave MacMillan. 10.1057/9781137007230

[B40] Mackay F. Kenny M. Chappell L. (2010). New Institutionalism through a Gender Lens: Towards a Feminist Institutionalism? Int. Polit. Sci. Rev. 31 (5), 573–588. 10.1177/0192512110388788

[B41] Madera J. M. Hebl M. R. Martin R. C. (2009). Gender and Letters of Recommendation for Academia: Agentic and Communal Differences. J. Appl. Psychol. 94 (6), 1591–1599. 10.1037/a0016539 19916666

[B42] Manfredi S. Grisoni L. Handley K. Nestor R. Cooke F. (2014). Gender and Higher Education Leadership: Researching the Careers of Top Management Programme Alumni. Research and Development Series. London: Leadership Foundation.

[B43] Martin P. Y. (2003). "Said and Done" versus "Saying and Doing". Gend. Soc. 17, 342–366. 10.1177/0891243203017003002

[B44] Martin P. Y. (2020). “Gendered Organizations: Fifty Years and Counting,” in Gender, Considered: Feminist Reflections across the US Social Sciences. Editors Fenstermaker S. Stewart A. (Cham Switzerland: Palgrave Macmillan), 263–296. 10.1007/978-3-030-48501-6_12

[B45] McIntosh P. (2010). Unpacking the Invisible Knapsack - National SEED Project on Inclusive Curriculum. Available at: https://nationalseedproject.org/Key-SEED-Texts/white-privilege-unpacking-the-invisible-knapsack.

[B46] Meyerson D. E. Scully M. A. (1995). Crossroads Tempered Radicalism and the Politics of Ambivalence and Change. Organ. Sci. 6 (5), 585–600. 10.1287/orsc.6.5.585

[B47] Misra J. Lundquist J. H. Holmes E. Agiomavritis S. (2011). The Ivory Ceiling of Service Work. Academe. 97 (1), 22–26.

[B48] Montez Lope E. O'Connor P. (2019). Micropolitics and Meritocracy: Improbable Bedfellows? Educ. Managment Adm. Leadersh. 47 (5), 678–693. 10.1177/1741143218759090

[B49] Morley L. (2006). Hidden Transcripts: The Micropolitics of Gender in Commonwealth Universities. Women's Stud. Int. Forum. 29 (1), 534–551. 10.1016/j.wsif.2006.10.007

[B50] Moscatelli S. Menegatti M. Ellemers N. Mariani M. G. Rubini M. (2020). Men Should Be Competent, Women Should Have it All: Multiple Criteria in the Evaluation of Female Job Candidates. Sex Roles. 83, 269–288. 10.1007/s11199-019-01111-2

[B80] Moss-Racusin C.A. Dovidio J.F. Brescoll V.L. Mark J. Graham M.J. Handelsman J. (2012). Faculty's subtle gender biases favor male students. Proceedings of the National Academy of Sciences 109 (41), 16474–16479. 10.1073/pnas.1211286109 PMC3478626 22988126

[B51] Nielsen M. W. (2016). Limits to Meritocracy? Gender in Academic Recruitment and Promotion Processes. Sci. Public Pol. 43 (3), 386–399. 10.1093/scipol/scv052

[B52] O'Connor P. Irvine G. (2020). Multi-level State Interventions and Gender Equality in Higher Education Institutions: The Irish Case. Administrative Sci. 10 (1), 98–119. 10.3390/admsci10040098

[B53] O'Connor P. (2014). Management and Gender in Higher Education. Manchester: Manchester University Press.

[B54] O'Connor P. Martin P. Y. Carvalho T. O'Hagan C. Veronesi L. Mich O. (2019). Leadership Practices by Senior Position Holders in Higher Educational Research Institutes: Stealth Power in Action? Leadership 15 (6), 722–743.

[B55] O'Connor P. O'Hagan C. (2016). Excellence in university Academic Staff Evaluation: a Problematic Reality? Stud. Higher Education 41 (11), 1943–1957. 10.1080/03075079.2014.1000292

[B56] O'Connor P. (2020a). Why Is it So Difficult to Reduce Gender Inequality in Male-Dominated Higher Educational Organisations? A Feminist Institutional Perspective. Interdiscip. Sci. Rev. 45 (2), 207–228. 10.1080/03080188.2020.1737903

[B57] O’Connor P. (2020b). “Accessing Academic Citizenship: Excellence or Micropolitical Practices?,” in Gendered Academic Citizenship: Issues and Experiences. Editor Sumer S. Palgrave, 37–64. 10.1007/978-3-030-52600-9

[B58] O’Connor P. (2019). An Autoethnographic Account of a Pragmatic Inclusionary Strategy and Tactics as a Form of Feminist Activism, *Equality, Diversity And Inclusion* . Int. J. 8, 825. 10.1177/1742715019853200

[B59] O’Connor P. Barnard S. (2021). “Problematising Excellence as a Legitimating Discourse,” in Gender, Power and Higher Education in a Globalised World. Editors O’Connor P. White K. (Palgrave Macmillan). (in press). 10.18556/touchsurgery/2021.s0180

[B60] O’Connor P. White K. (2021). “Power, Legitimating Discourses and Institutional Resistance to Gender equality in Higher Education,” in Gender, Power and Higher Education in a Globalised World. Editors O’Connor P. White K. (Palgrave Macmillan). (in press).

[B61] Oswald F. L. Mitchell G. Blanton H. Jaccard J. Tetlock P. E. (2015). Using the IAT to Predict Ethnic and Racial Discrimination: Small Effect Sizes of Unknown Societal Significance. J. Personal. Soc. Psychol. 108 (4), 562–571. 10.1037/pspa0000023 25844574

[B62] Peterson H. Carvalho T. Jordansson B. de Lourdes Machado-Taylor M. (2021). “Institutionalised Resistance to Gender equality Initiatives in Swedish and Portuguese Academia,” in Gender, Power and Higher Education in a Globalised World. Editors O’Connor P. White K. (Palgrave Macmillan). (in press).

[B63] Powell S. Ah-King M. Hussénius A. (2018). 'Are We to Become a Gender university?' Facets of Resistance to a Gender equality Project. Gend. Work Organ. 25 (2), 127–143. 10.1111/gwao.12204

[B64] Rorstad K. Aksnes D. W. (2015). Publication Rate Expressed by Age, Gender and Academic Position – A Large-Scale Analysis of Norwegian Academic Staff. J. Infometrics. 9 (2), 317–333. 10.1016/j.joi.2015.02.003

[B65] Rudman L. A. Glick P. (2001). Prescriptive Gender Stereotypes and Backlash toward Agentic Women. J. Soc. Isssues. 57 (4), 743–762. 10.1111/0022-4537.00239

[B66] Saglamer G. Tan M. Caglayan H. Almgren N. Salminen-Karlsson M. Baisner L. (2016). FESTA Handbook on Resistance to Gender Equality in Academia. Available at: https://www.festa-europa.eu/sites/festa-europa.eu/files/FESTA%20D7.1%20Handbook%20on%20Resistance%20to%20Gender%20Equality%20in%20Academia.pdf (Accessed June 28, 2021)

[B82] Schein E. H. (1988). Innovative Cultures and Organsiations. Massachusetts Institute of Technology Available at: https://dspace.mit.edu/bitstream/handle/1721.1/2214/SWP-2066-21290193.pdf?sequence=1/

[B67] Simons H. (2009). Case Study Research in Practice. Los Angeles: Sage. 10.4135/9781446268322

[B68] Sinclair A. (2005). Doing Leadership Differently: Gender, Power, and Sexuality in a Changing Business Culture. 2^nd^ ed. Melbourne: Melbourne University Press.

[B79] Smith J. Noble H. (2014). Bias in Research. Evid.-Based Nurs. 17, 100–101. 10.1136/eb-2014-101946 25097234

[B69] Smolovic Jones O. Smolovic Jones S. Taylor S. Yarrow E. (2020). I Wanted More Women in, but . . .’: Oblique Resistance to Gender Equality Initiatives. Work Employ. Soc. 10.1177/0950017020936871

[B78] Stratigaki M. (2005). Gender mainstreaming vs. Positive Action: An ongoing conflict in EU Gender Equality Policy. Eur. J. Women’s Stud. 12 (2), 165–186.

[B70] Temitope Igiebor O. (2021). Gender Equity Policy and Women in Academic Leadership Positions in Nigeria. New Zealand: PhD thesis submitted to the University of Auckland.

[B71] Trix F. Psenka C. (2003). Exploring the Color of Glass: Letters of Recommendation for Female and Male Medical Faculty. Discourse Soc. 14 (2), 191–220. 10.1177/0957926503014002277

[B72] Tyler T. (2005). Introduction: Legitimating Ideologies. Soc. Justice Res. 18, 210–215. 10.1007/s11211-005-6822-4

[B73] Van den Brink M. Benschop Y. Jansen W. (2010). Transparency in Academic Recruitment: A Problematic Tool for Gender Equality? Organ. Stud. 31 (11), 1459–1483. 10.1177/0170840610380812

[B74] Van den Brink M. Benschop Y. (2012). Slaying the Seven-Headed Dragon: The Quest for Gender Change in Academia. Gend. Work Organisation. 19 (1), 71–92. 10.1111/j.1468-0432.2011.00566.x

[B75] Van den Brink M. (2015). The Politics of Knowledge: the Responses to Feminist Research from Academic Leaders. Int. J. 34 (6), 483–495. 10.1108/edi-01-2015-0004

[B76] Webb P. T. (2008). Re‐mapping Power in Educational Micropolitics. Crit. Stud. Education. 49 (2), 127–142. 10.1080/17508480802040183

